# Importance of Video-EEG Monitoring in the Diagnosis of Epilepsy in a Psychiatric Patient

**DOI:** 10.1155/2013/159842

**Published:** 2013-03-25

**Authors:** Batool F. Kirmani

**Affiliations:** Epilepsy Center, Department of Neurology, Scott & White Neuroscience Institute and Texas A&M Health Science Center College of Medicine, Temple, TX 76508, USA

## Abstract

Epilepsy is a chronic medical condition which is disabling to both patients and caregivers. The differential diagnosis of epilepsy includes psychogenic nonepileptic spells or “pseudoseizures.” Epilepsy is due to abnormal electrical activity in the brain, and pseudoseizure is a form of conversion disorder. The brain waves remain normal in pseudoseizures. The problem arises when a patient with significant psychiatric history presents with seizures. Pseudoseizures become high on the differential diagnosis without extensive work up. This is a case of woman with significant psychiatric issues which resulted in a delay in the diagnosis of epilepsy.

## 1. Case Report

Ms. V is a 33-year-old married right-handed Caucasian woman with history of questionable spells characterized by loss of consciousness and whole body jerking since the age of 13. She had several EEGs in the past which were normal. She has a history of significant anxiety issues including depression and behavioral problems. She attributes most of her problems to childhood abuse. There have been instances in the emergency room where there has been a delay in treatment because of high suspicion of pseudoseizures. Her first episode of generalized body jerking was at the age of 13, with a second episode one year later. She was initially prescribed Dilantin and then phenobarbital by a local physician. She could not tolerate these medications and continued to be intractable. The EEGs were also negative. However, she never had a prolonged EEG under medication withdrawal where the spell was actually captured. She was started on Depakote at age 14, which did improve the spells but it was tapered off because of weight gain. She was switched to Topamax with no effective control. She was on a combination of Tegretol and Lamictal for a year when she was seen in our epilepsy clinic. She was having several spells with associated injuries and at least 1-2 emergency room visits every month or every other month. She also had isolated auras of deja vu sensation at least twice a week, which lasted for about a minute without any alteration of consciousness. She denies any family history of epilepsy, febrile seizures, TIA, stroke, or meningitis. She has had an abusive childhood and has had several domestic issues going on with her children and siblings. Her examination was completely normal. She was admitted to the epilepsy monitoring unit in order to capture her spells for definitive diagnosis. Lamictal and Tegretol were withdrawn but unfortunately no spells were captured and her EEG was normal. Based on the description of the spells, she was discharged on Lamictal and Keppra. Tegretol was discontinued during this stay. She continued to have breakthrough spells and Keppra aggravated the mood issues.

There was always a bias in terms of her psychiatric history that she may be having pseudoseizures. She was brought again to the epilepsy monitoring unit a few months later to capture her spells. Lamictal and Keppra were withheld the day of admission. She had one spell lasting for 2 minutes characterized by whole body jerking and loss of consciousness. This was associated with a generalized spike and wave discharge consistent with generalized epilepsy. She was restarted on Depakote for generalized epilepsy and mood disorder since she had had a favorable trial in terms of spells. She has been essentially seizure free for the last 2 years with occasional breakthrough if she forgets to take her medication.

This case report emphasizes the need for specialized testing when establishing a diagnosis. In this case, it took almost two decades for an accurate diagnosis of generalized epilepsy to be made. Seizures are due to hyperactivity of the cortical neurons or synchronous neuronal activity in the brain [1]. In primary generalized epilepsy, the abnormal electrical activity starts simultaneously from both the right and the left cerebral hemispheres [2]. The diagnosis of epilepsy includes a careful history and EEG findings. Her history was consistent with generalized epilepsy. She does have auras which are commonly considered a part of localization related to epilepsy. However, it has been shown in the literature that 70% of patients with generalized epilepsy also have auras [3]. It has been shown that patients with epilepsy do have other psychiatric comorbidities. The most common psychiatric conditions in epilepsy are anxiety, depression, and psychoses [4]. So epilepsy should not be excluded in these subsets of patients. Pseudoseizures are also high on the differential diagnosis because of her abusive childhood and stressful life but should not be considered as the primary diagnosis. Pseudoseizures fall under the category of somatoform disorders according to the Diagnostic and Statistical Manual of Mental Diseases. Somatoform disorders involve unconscious manifestation of the physical symptoms due to internal issues, which the patient is unable to express. These physical symptoms produced by the patient are not under voluntary control. The main focus of this paper is to establish diagnosis in order to find the right treatment for these patients and to avoid medicines which decrease the seizure threshold. Our patient was started on Depakote which provides good seizure control against generalized epilepsy and also works well with psychiatric comorbidities [5]. 
